# Supplementary figures and images for: Underwater light climate and wavelength dependence of microalgae photosynthetic parameters in a temperate sea
Source: PeerJ. 2021 Oct 4;9:e12101. doi: 10.7717/peerj.12101 (PMC8496463; doi:10.7717/peerj.12101)

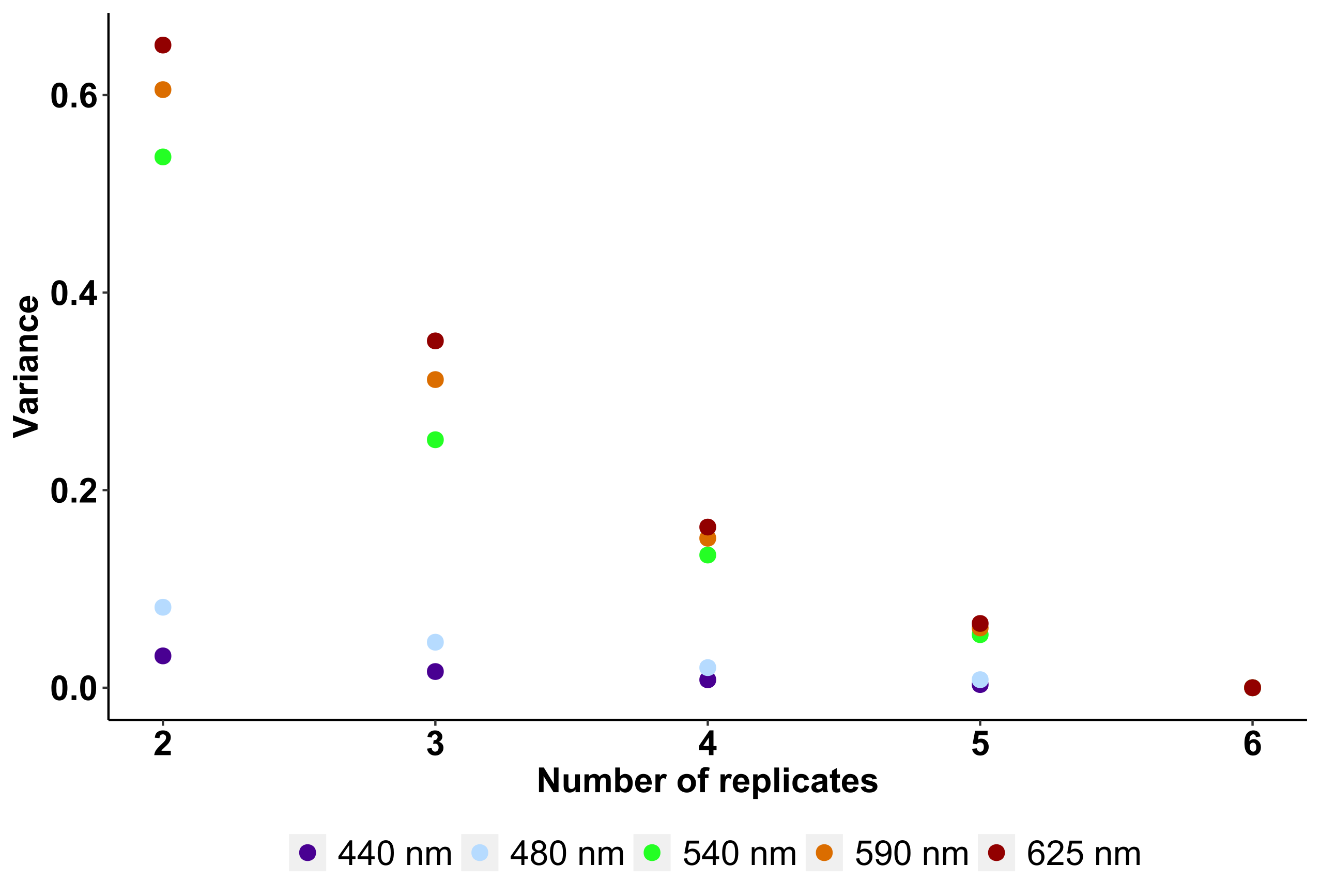

Supplement: Supplemental Information 1 — Variance of the functional absorption cross section of photosystem II (Sigma(II)λ) by wavelength as a function of the number of replicates. See Schreiber, Klughammer & Kolbowski (2012) for more details about Sigma(II)λ and protocol measurements with the MULTI-COLOR-PAM (Heinz Walz GmbH, Germany). [file peerj-09-12101-s001.png]

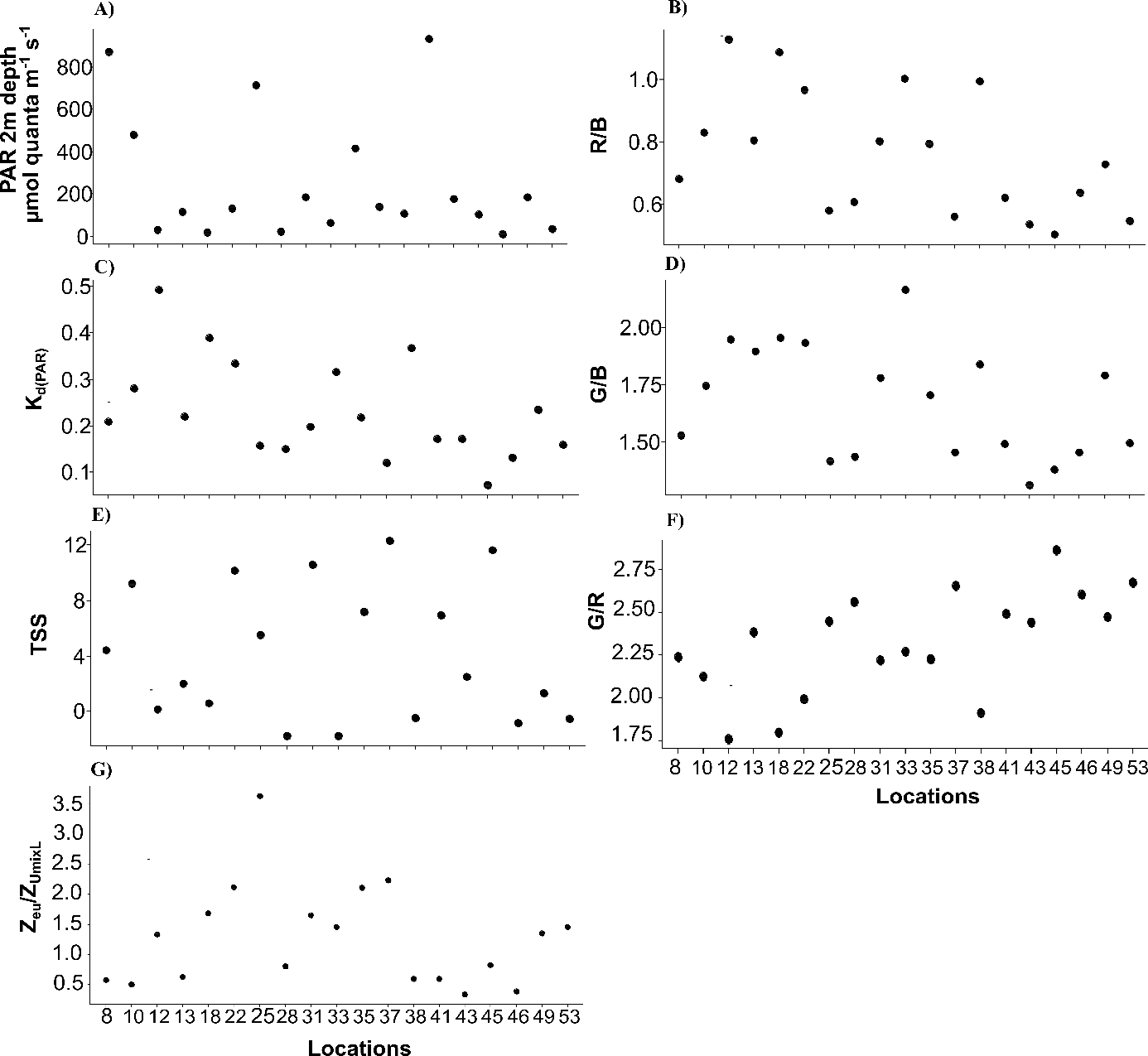

Supplement: Supplemental Information 3 — Variables related to the light climate of the 19 locations: A) photosynthetically active radiation (PAR) at a depth of 2 m; (C) Kd(PAR); (E) time since sunrise (h) (TSS); and light-quality ratios at the surface of (B) (R)ed/(B)lue, (D) (G)reen/(B)lue and (F) (G)reen/(R)ed wavelengths. [file peerj-09-12101-s003.png]

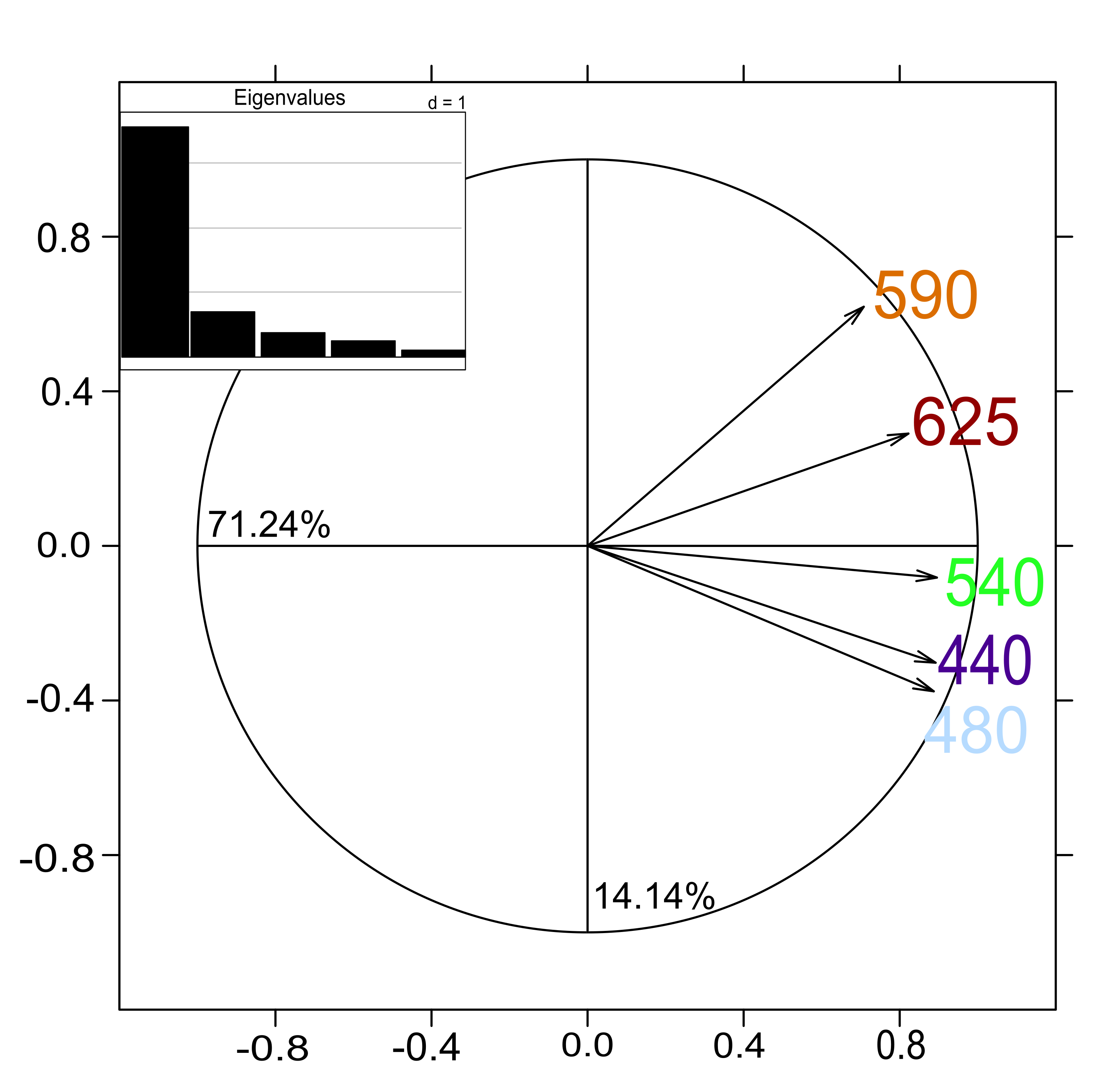

Supplement: Supplemental Information 5 — Correlation plot (first and second dimension) of interstructure results of partial triadic analysis interstructure analysis of detrended photosynthetic parameters measured at 440, 480, 540, 590 and 625 nm and eigenvalues values histogram. [file peerj-09-12101-s005.png]

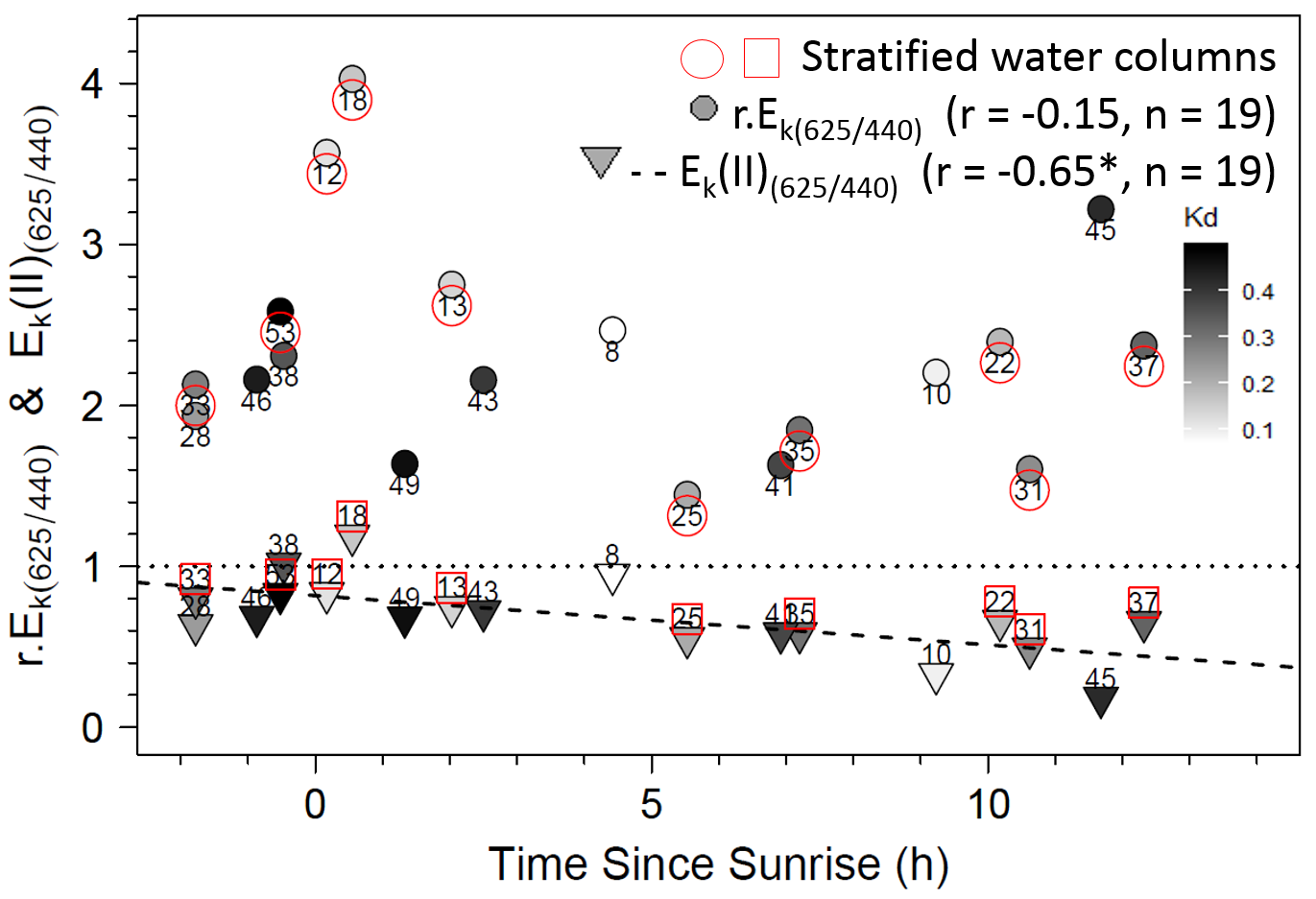

Supplement: Supplemental Information 6 — Relationships between the Ek,625/440 ratio (ratio of photoacclimation index measured at red and blue wavelengths) in relative(r) (circles) and absolute (II) (inversed triangles) units and TSS (Time since sunrise) in water masses for the 19 locations. Red circles and squares indicate samples with stratified water columns. Gray scale color refers to Kd values. The regression equation is y = −0.0306 x + 0.8189 (F = 12.41; p =0.0026). Pearson correlation coefficients, level of significance (* p < 0.05) and the number of considered data are also reported on each graph. [file peerj-09-12101-s006.png]

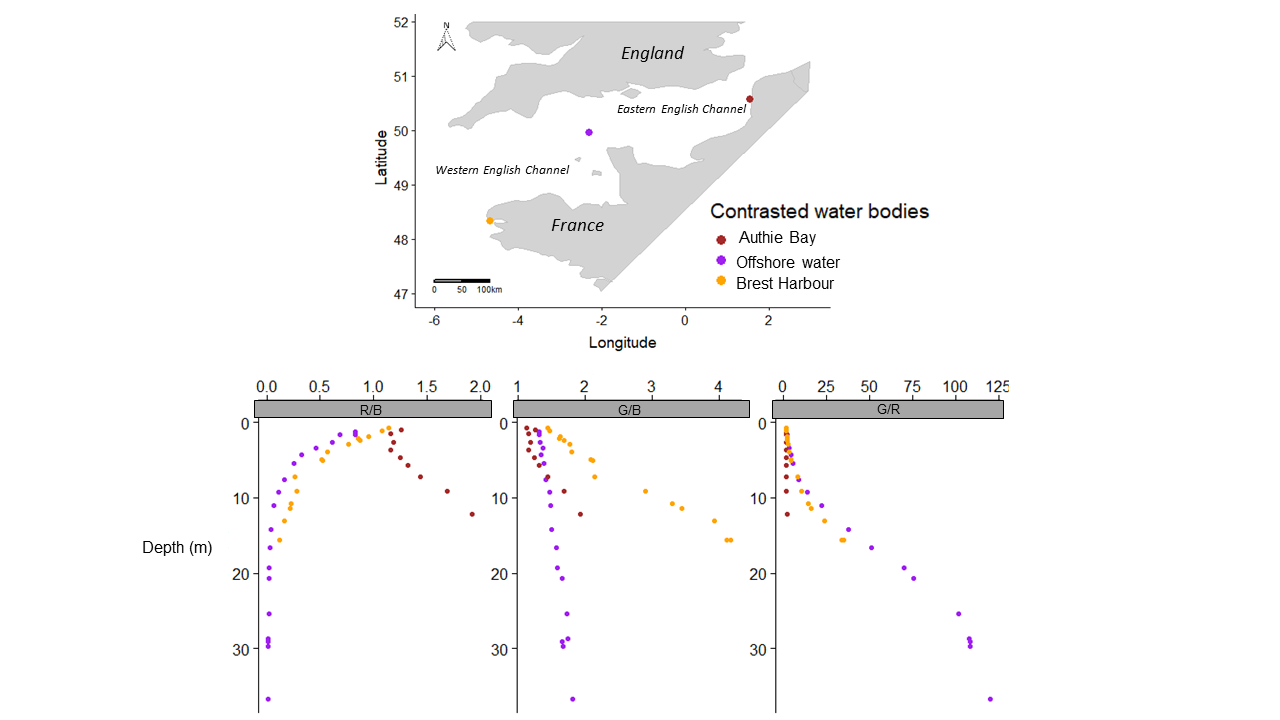

Supplement: Supplemental Information 7 — Vertical profile of light quality ratios of three different water masses in the coastal area of the English Channel during the sampling cruise (map in the top of the figure) i) Authie Bay (brown circles) ii) the offshore sample close to Jersey Islands (purple circles) and iii) in the Brest Harbor (orange circles). In the bottom of the figure it is shown the vertical profiles of red/blue (R/B), green/blue (G/B) and green/red (G/R) light ratio for each water mass. [file peerj-09-12101-s007.png]
